# Comparison of Human Induced Pluripotent Stem-Cell Derived Cardiomyocytes with Human Mesenchymal Stem Cells following Acute Myocardial Infarction

**DOI:** 10.1371/journal.pone.0116281

**Published:** 2014-12-31

**Authors:** Lucas Citro, Shan Naidu, Fatemat Hassan, M. Lakshmi Kuppusamy, Periannan Kuppusamy, Mark G. Angelos, Mahmood Khan

**Affiliations:** 1 Davis Heart and Lung Research Institute, The Ohio State University, Columbus, Ohio, 43210, United States of America; 2 Division of Cardiovascular Medicine, The Ohio State University, Columbus, Ohio, 43210, United States of America; 3 Department of Emergency Medicine, The Ohio State University, Columbus, Ohio, 43210, United States of America; 4 Department of Radiology, Geisel School of Medicine at Dartmouth, Dartmouth College, New Hampshire, 03766, United States of America; University of Central Florida, United States of America

## Abstract

**Introduction:**

Human induced pluripotent stem cell-derived cardiomyocytes (hiPSC-CMs) have recently been shown to express key cardiac proteins and improve *in vivo* cardiac function when administered following myocardial infarction. However, the efficacy of hiPSC-derived cell therapies, in direct comparison to current, well-established stem cell-based therapies, is yet to be elucidated. The goal of the current study was to compare the therapeutic efficacy of human mesenchymal stem cells (hMSCs) with hiPSC-CMs in mitigating myocardial infarction (MI).

**Methods:**

Male athymic nude hyrats were subjected to permanent ligation of the left-anterior-descending (LAD) coronary artery to induce acute MI. Four experimental groups were studied: 1) control (non-MI), 2) MI, 3) hMSCs (MI+MSC), and 4) hiPSC-CMs (MI+hiPSC-derived cardiomyocytes). The hiPSC-CMs and hMSCs were labeled with superparamagnetic iron oxide (SPIO) *in vitro* to track the transplanted cells in the ischemic heart by high-field cardiac MRI. These cells were injected into the ischemic heart 30-min after LAD ligation. Four-weeks after MI, cardiac MRI was performed to track the transplanted cells in the infarct heart. Additionally, echocardiography (M-mode) was performed to evaluate the cardiac function. Immunohistological and western blot studies were performed to assess the cell tracking, engraftment and cardiac fibrosis in the infarct heart tissues.

**Results:**

Echocardiography data showed a significantly improved cardiac function in the hiPSC-CMs and hMSCs groups, when compared to MI. Immunohistological studies showed expression of connexin-43, α-actinin and myosin heavy chain in engrafted hiPSC-CMs. Cardiac fibrosis was significantly decreased in hiPSC-CMs group when compared to hMSCs or MI groups. Overall, this study demonstrated improved cardiac function with decreased fibrosis with both hiPSC-CMs and hMSCs groups when compared with MI group.

## Introduction

Myocardial infarction (MI) involves the death or damage of the myocardium [1]. Approximately 1,450,000 Americans suffer from an MI each year [2], with thirty five to forty percent of all acute myocardial infarctions producing fatal outcomes [3]. Stem cell-based cardiac therapy has been proposed as a viable candidate to mitigate cardiomyocyte loss and impaired cardiac function subsequent to a MI and thus improve patient prognosis. Many cell types, including mesenchymal stem cells [4], skeletal myoblasts [5], embryonic stem cells [6], and cardiospheres [7] have been investigated in both pre-clinical and clinical MI studies to evaluate their potential efficacy in improving cardiac function and regenerating necrotic myocardium. In a recent murine study by Laflamme et al., administration of human embryonic stem cell-derived cardiomyocytes (hESCs) prevented cardiac functional loss between 48 hours and four weeks following MI, as determined from the fractional shortening of treated animals [6]. However, the possibility of teratoma formation [8], in addition to a negative ethical stigma, limits the full realization of potential hESC-based therapies [8], [9].

Recent groundbreaking work in the field of stem cell research by Takashi and Yamanaka demonstrated that mouse fibroblasts can be reprogrammed by ectopic expression of four transcription factors, *Oct4*, *Klf4*, *Sox2*, and c-*Myc* to induced pluripotent stem (iPS) cells [10]. Induced pluripotent stem cells (iPSC) possess many of the distinguishing qualities characteristic of embryonic stem cells (ESC), such as unlimited replication [11] and pluripotency, without the negative connotations associated with ESC research [12]. In addition, the possibility for the immune rejection of transplanted cells may be greatly reduced as the iPS cells can be derived from autologous patient-specific cells [11]. IPS cells can be generated by “reprogramming” cells from various unique sources, including human dermal fibroblasts [12] and skeletal myoblasts [13], using viral gene transduction [14].

Although iPS cells can be used in their native, undifferentiated form, such use has been shown to promote tumor genesis in control and MI rats regardless of the administered dose [15]. To avoid tumor genesis, and thereby improve therapeutic potential, iPS cells may be differentiated to a particular lineage, such as endothelial [16] or cardiomyocyte [17] prior to *in vivo* use. Cardiac progenitor cells, generated from hiPS cells, have been shown to express cardiac proteins essential to the development of an adult ventricular myocyte phenotype, such as connexin-43 and myosin chain complexes, when cultured in vitro [18]. Similarly, cardiomyocytes differentiated from mouse skeletal myoblast-derived iPS cells have been shown to form fully-developed, spontaneously-beating sarcomeres when cultured *in vitro* [19]. Through examination of action potential characteristics, iPS-derived cells have likewise demonstrated sensitivity to β-adrenergic stimulation and differentiation potential for ventricular, atrial, and nodal cardiomyocyte lineages [20]. When administered *in vivo*, iPS-derived cardiomyocytes have been shown to effectively integrate with host myocardium and thus significantly decrease fibrosis while significantly increasing fractional shortening in a murine model of MI [17]. In a study by Pasha et al., a significant decrease in cardiac fibrosis and improvement in cardiac function were observed in post-MI mice treated with cardiac progenitors differentiated from skeletal myoblast-derived iPS cells (SiPS-CP) [13]. In addition, mouse iPSC-derived myocardial tissue sheets, known as bioengineered myocardium, have recently been shown to increase cardiac contractility, relative to No-MI animals, four weeks following MI [21].

In recent years, both preclinical and clinical studies have demonstrated attenuation of acute MI injury with MSC therapy [22], [23]. Based on our previously published work on MSCs and myocardial repair, we have chosen hMSCs as a control to compare with hiPSC-CMs in attenuating MI [22], [23]. Therefore, the main objective of this study was to compare the effect of hiPS-CM with hMSC transplantation, on recovery of cardiac function in a rat model of acute MI. To our knowledge this is the first study to directly compare these two different types of stem cells for cardiac therapy. Our results demonstrate that hiPSC-CMs are equipotent with hMSCs in improving cardiac function following MI. Engrafted hiPSC-CMs cells expressed cardiomyocyte markers and decreased cardiac fibrosis in the infarct heart when compared with hMSCs. No tumors or arrhythmias were observed in hiPSC-CMs or hMSCs transplanted heart.

## Materials and Methods

### Culturing and labeling of stem cells with SPIO for tracking their engraftment in the heart

Human inducible pluripotent stem cells (hiPSC-CMs) were procured from CDI (Cellular Dynamics International; Madison, Wisconsin). These cells expressed enhanced red fluorescence protein (RFP) for *in-vivo* tracking. The hiPSC-CMs were cultured in the plating media provided by CDI for 48 h in a T75 flask and incubated at 37°C, with a mixture of air and 5% CO_2_ in a humidified chamber. After 2 days of culture, 10 mL of pre-warmed fresh maintenance growth media was added to each flask. The maintenance culture media was changed every 2–3 days. Human iPSC-CMs were beating spontaneously at one week after culture (**S1 Video**).

Human MSCs were acquired from Lonza (***Lonza Biologics Inc***, Portsmouth NH). hMSCs were cultured in a T75 flask containing MSC plating media provided by Lonza and incubated at 37°C, with a mixture of air and 5% CO_2_ in a humidified chamber. hMSCs were cultured until they reached 80–90% confluency. The cell culture media was replaced every 2–3 days. Both hiPSC-CMs and hMSCs were incubated with super paramagnetic iron oxide (SPIO) particles (0.9 µm diameter, 1.25×10^5^/mL medium; Bangs Laboratories, IN, USA) for 24 hours. The SPIO particles used during this study were labeled with Dragon-green fluorophores. To remove un-internalized SPIO particles, cells were rinsed three times with PBS and placed in fresh media. Using a series of three intramyocardial injections, hMSCs and hiPSC-CMs (1×10^6^ cells total, in 100 µL serum-free medium) were transplanted into the infarct and peri-infarct regions of the left ventricular myocardium thirty minutes following permanent LAD coronary artery ligation.

### Expression of cardiac markers by hiPSC-CMs in vitro

Immunofluorescence staining was performed in cells or cardiac sections fixed with paraformaldehyde. The fixed cells were washed with PBS and then incubated with 2% goat serum and 5% bovine-serum albumin in PBS to reduce nonspecific binding. The cells or cardiac sections were then incubated for 2 hours at room temperature with mouse, anti-α-sarcomeric actinin (1∶500, Sigma-Aldrich, MO), cardiac troponin-T (1∶200, Santa Cruz, CA), myosin heavy chain (1∶200, Santa Cruz, CA) and connexin-43 monoclonal antibodies (1∶500, Cell Signaling, MA). The sections were then incubated with the appropriate anti-mouse secondary antibodies (1∶1000) conjugated to Texas red and FITC. The nuclei were counterstained with HardSet mounting medium with DAPI (Vector Labs). The cells were visualized by inverted Nikon fluorescence microscope (TE 2000). Separate cells were also stained without primary antibodies to identify nonspecific binding.

### Induction of myocardial infarction

**Figure 1:**
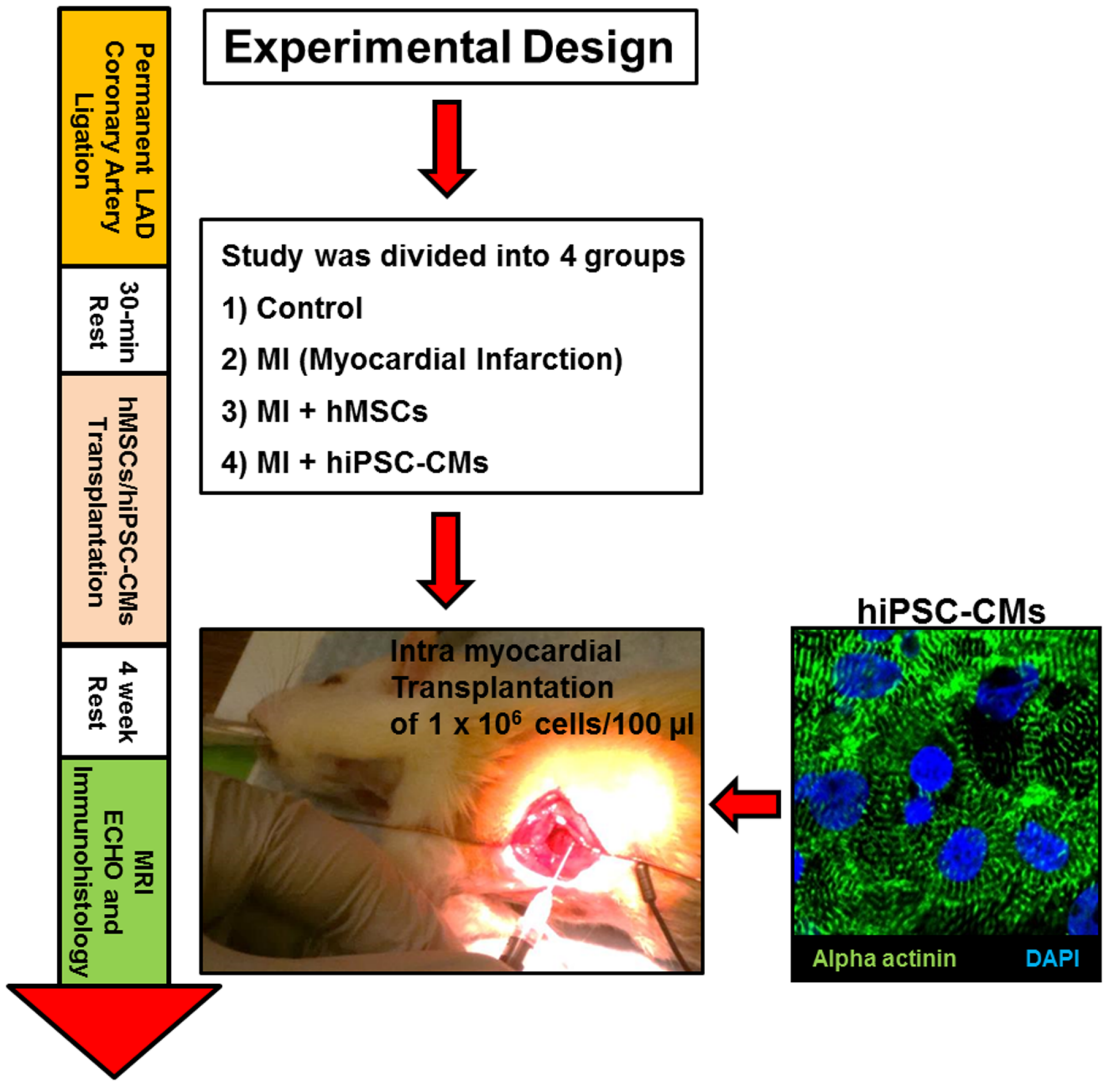
Experimental Design. Schematic representation of experimental design, indicating relative timing of LAD coronary artery ligation, stem cell administration, and functional analyses.

Male athymic nude rats (Hsd∶RH-Foxn1^rnu^, Harlan Laboratories, Greenfield, IN), 200–250 grams were randomly separated into four groups (**Fig. 1**, n = 6/group) as following: (i) Control group (No-MI); (ii) MI group (treated with serum-free medium); (iii) hMSCs group (MI treated with hMSCs transplantation); (iv) hiPSC-CMs group (MI treated with hiPSC-CMs). Rats were anesthetized initially with ketamine (50 mg/kg; I.P.) and xylazine (5 mg/kg; I.P.) and maintained using a 1.5–2.0% isoflurane. Anesthesia was confirmed by the absence of the pedal reflex. MI was induced by permanently ligating the LAD coronary artery. The LAD ligation was standardized in all similar sized animals by ligating the LAD 3-mm distal to the left atrium. Complete ligation is assessed by the rapid change in the color, pallor and akinetic movement of the distal LAD perfused myocardium. ST elevation was observed with continuous ECG monitoring obtained during the procedure. The chest was closed and animals were extubated and removed from anesthesia following restoration of spontaneous respiration as published [4], [22], [23].

### Ethics

All animal procedures performed during this study were approved by the Institutional Animal Care and Use Committee of The Ohio State University and complied with the Guide for the Care and Use of Laboratory Animals (NIH Publication No. 86-23).

### Cardiac magnetic resonance imaging (MRI) for non-invasive tracking of cell engraftment in vivo

Cardiac magnetic resonance imaging (MRI) was performed using a Bruker 9.4T horizontal-bore imaging system equipped with Paravison 4.0 (Bruker BioSpin, Billerica, MA, USA) as published earlier [22]. Cardiac function was analyzed four weeks following hiPSC-CMs and hMSCs transplantation. Rats were anaesthetized initially with 1.5–2% isoflurane mixed with carbogen (1 L/min). Utilizing an abdominal pneumatic pad and electrode pads placed on the forepaw and foot of each animal, physiologic parameters such as the EKG and respiration were monitored using an MRI-compatible system (Model 1025, Small Animal Instruments, Stonybrook, NY, USA). Each animal was secured to a water-heated animal bed and placed near the isocenter of the MRI scanner. Cardiac gated, short-axis T1-weighted (T1W) images were acquired in order to cover the entire left ventricle of each rat (FLASH-cine sequence parameters: TR = 16 ms; TE = 1.6 ms; α = 10°; matrix = 256×192; FOV = 5.1×5.1 cm; slice thickness = 2 mm, N = 8, movie cycles adjusted based on animal heart rate). The length of each imaging session was between 45 and 90 minutes [22].

### M-mode echocardiography

M-mode echocardiography was also performed to assess cardiac functional recovery following infarction. During imaging, animals were anesthetized using 1.5–2.0% isoflurane in air. Echocardiography was performed 4 weeks following hiPSC-CMs and hMSCs transplantation using a high-resolution Vevo-2100 ultrasound imaging system (Visualsonics; Toronto, Canada) as published earlier [23]. Echocardiographic measurements were taken utilizing both long and short axes B and M-mode views. The B-mode cuts are used to standardize the anatomic location of the M-mode acquisition in the heart.

### Engraftment of transplanted hiPSC-CMs in the heart

At four weeks after MI, hiPSC-CMs engraftment was assessed using fluorescence microscopy. Transplanted hiPSC-CMs were identified in the LV myocardial sections by RFP fluorescence and by SPIO dragon green fluorescence as published previously [23].

### Expression of cardiac markers in the engrafted hiPSC-CMs in the infarct heart

Immunofluorescence staining with primary antibodies against α-actinin, myosin heavy chain and conexin-43 was performed as mentioned above [23].

### Assessment of cardiac fibrosis

Rats were anaesthetized after 4 weeks following MI or stem cell transplantation, and their hearts were excised and washed with ice-cold PBS. The hearts were then frozen for 10-minutes at −20°C and sliced into 2 mm sections using a heart matrix. The LV myocardial sections were then incubated overnight in formalin to perform masson-trichrome staining for assessment of cardiac fibrosis. To determine the fibrosis, images were acquired by a dissecting microscope (Nikon; Tokyo, Japan). The fibrosis area (Blue color) was expressed as a percentage of total LV area and quantified as by computerized planimetry using MetaMorph image analysis software (Molecular Devices; Sunnyvale, CA) as described previously [23].

### Data analysis

The statistical significance of the results was evaluated by one-way analysis of variance (ANOVA) and all pairwise multiple comparison procedures were done by Tukey's Post Hoc Test. The values were expressed as mean ± S.D. A p value of <0.05 was considered statistically significant.

## Results

### Expression of cardiac markers by hiPSC-CMs in vitro

**Figure 2:**
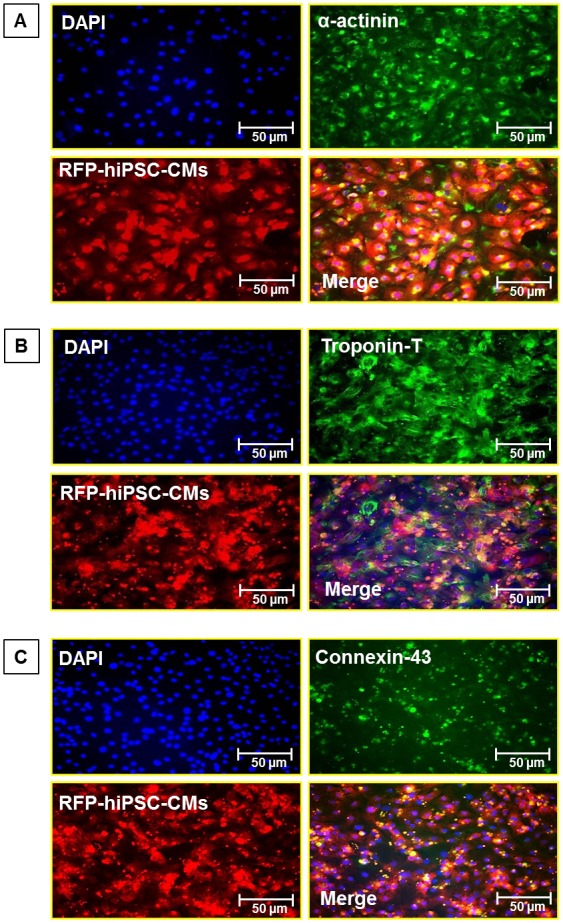
In-vitro expression of cardiac markers in hiPSC-CMs. Immunohistological staining shows expression of (**A**) α-actinin, (**B**) Troponin-T and (**C**) Connexin-43, by human iPSC-derived cardiomyocytes. RFP (red fluorescence protein, red color); DAPI (blue color).

Immunofluorescence studies showed that hiPSC-CMs express cardiomyocyte markers including α-actinin, troponin-T and conexin-43 *in vitro* (**Fig. 2**). Additionally these cardiomyocytes started to beat spontaneously at one-week after cell culturing (**S1 Video**).

### Assessment of cardiac function by M-mode echocardiography

**Figure 3:**
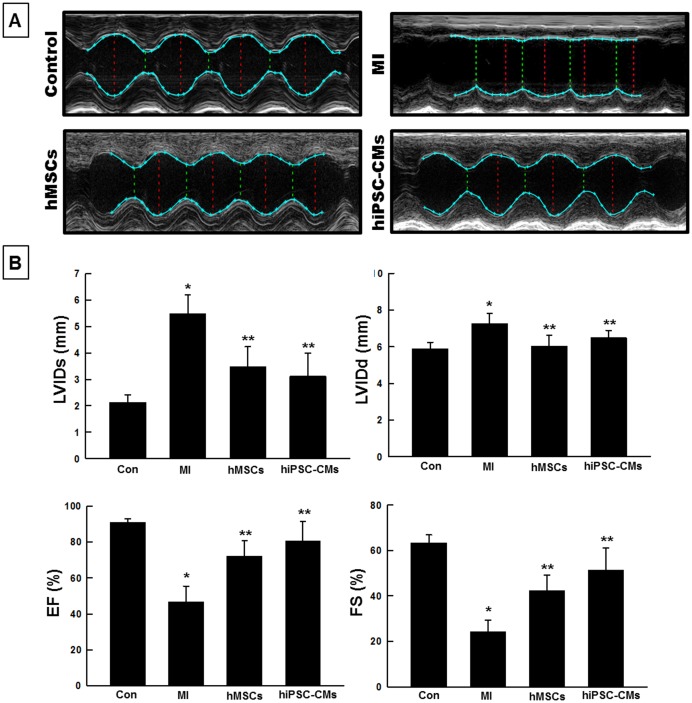
Cardiac functional recovery following hiPSC-CMs and hMSCs transplantation. (**A**) Transthoracic M-mode echocardiography images indicating recovery of ventricular wall movement for the hiPSC-CMs hearts. (**B**) LV functional data acquired four weeks following myocardial infarction. Significant improvements in Left ventricular internal diameter at systole (LVIDs), Left ventricular internal diameter at Diastole (LVIDd) ejection fraction (EF%) and fractional shortening (FS%) were observed in hiPSC-CMs and hMSCs groups, relative to the MI group. Although hiPSC-CM showed superior improvement in cardiac function, no significant difference was observed between hiPSC-CMs and hMSCs groups. All values expressed as Mean ± SD (n = 5). *p<0.05 vs. control; **p<0.05 vs. MI.

Cardiac function was evaluated by using transthoracic M-mode echocardiography at four weeks following myocardial infarction (**Fig. 3A**). The LV ejection fraction (EF) and fractional shortening (FS) significantly improved in both hMSCs and hiPSC-CMs groups compared to the MI group and although not statistically significant, hiPSC-CMs showed a trend of superior improvement in cardiac function when compared to the hMSCs group (**Fig. 3B**). We did not observe any significant difference in cardiac function at baseline (pre-MI) among different groups.

### Cardiac MR imaging for engraftment of hiPSC-CMs and hMSCs in the infarct heart

**Figure 4:**
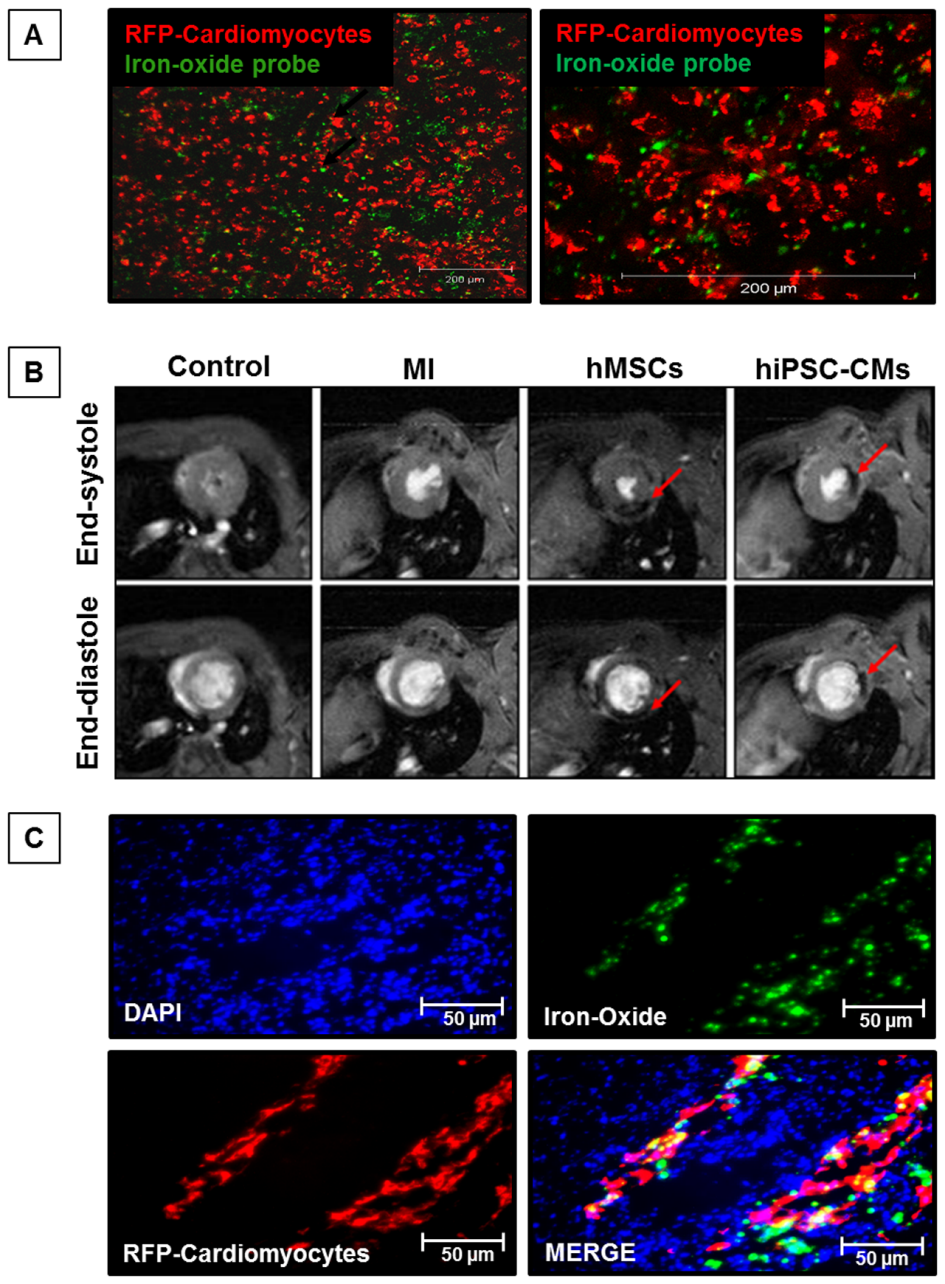
*In vivo* tracking of SPIO-labeled hiPSC-CMs and hMSCs in the infarct heart at four weeks following MI. (**A**) ***In vivo*** images of hiPSC-CMs labeled with SPIO particles. (**B**) Transaxial T1W FLASH-cine MR images acquired four weeks following MI using a horizontal bore 9.4T MRI system. The dark, hypodense regions, indicated by arrows, represent the presence of SPIO-labeled hiPSC-CMs in the LV myocardium of the athymic nude rats (n = 3–4). (**C**) Immunohistochemistry of LV cardiac tissue shows engraftment of RFP transfected hiPSC-CMs in the infarct heart 4 weeks following MI. Additionally, Merge image shows green fluorescence in the engrafted hiPSC-CMs labeled with SPIO.

The iron oxide labeled hiPSC-CMs and hMSCs (**Fig. 4A**) were visualized in the LV of the infarct myocardium by cardiac MR imaging at both end-systole and end-diastole (**Fig. 4B**). The hypointense regions of the LV myocardium indicated the engraftment of transplanted iron oxide-labeled hiPSC-CMs and hMSCs (**Fig. 4B**). MR images confirmed the engraftment of stem cells in the infarct myocardium at four weeks after MI. Similarly, Immunohistology results further confirmed the engraftment of transplanted hiPSC-CMs in the infarct heart as indicated by RFP and dragon green fluorescence in the LV area (**Fig. 4C**).

### Attenuation of cardiac fibrosis by hiPSC-CMs transplantation

**Figure 5:**
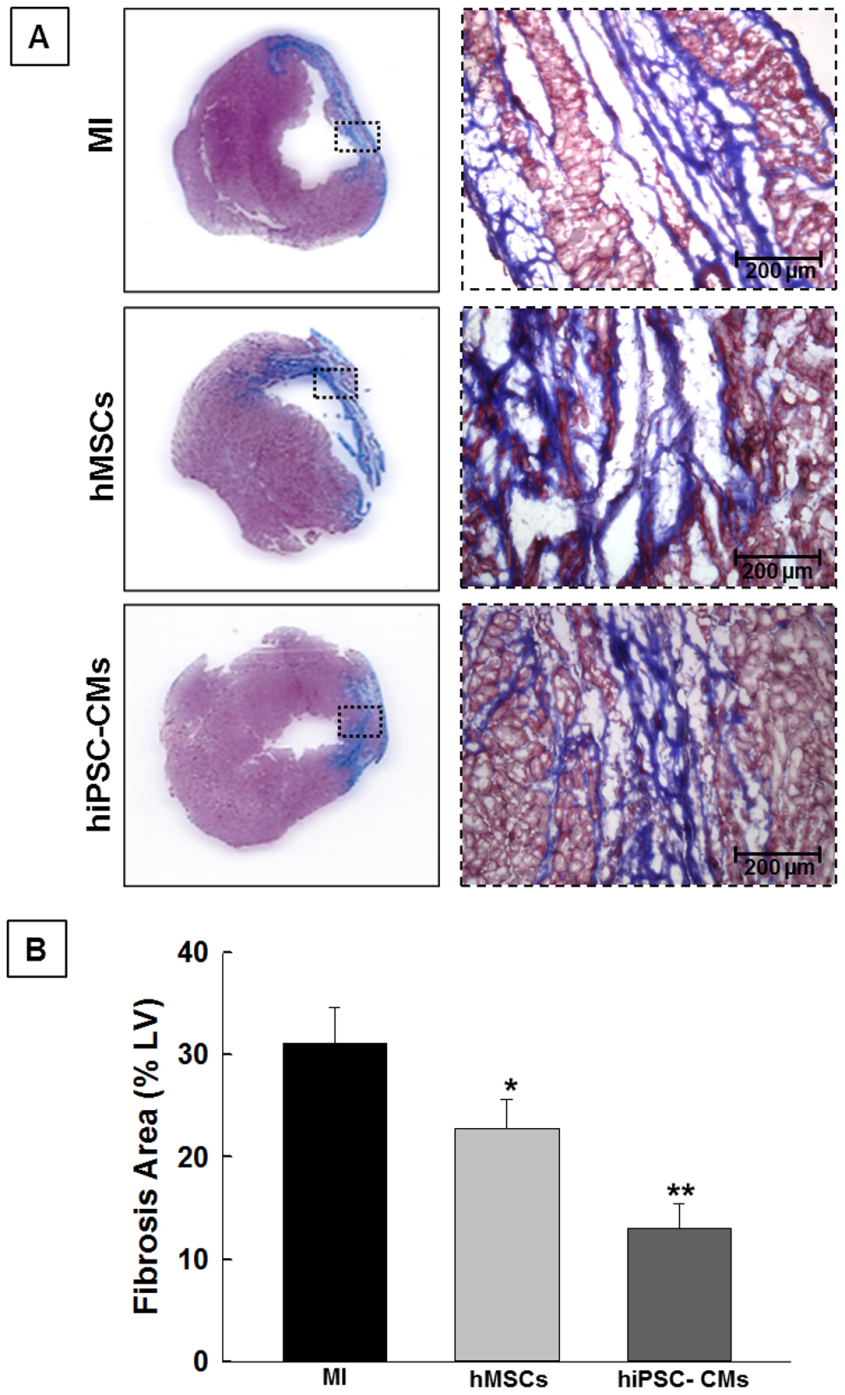
Assessment of cardiac fibrosis in the stem cell transplanted heart. (**A**) Representative images of Masson-trichrome staining of the heart indicating cardiac fibrosis (blue color) in MI, hMSCs and hiPSC-CMs groups at 4 weeks following MI. (**B**) Cardiac fibrosis was significantly attenuated in the hiPSC-CMs group compared to hMSCs or MI groups. All values expressed as Mean ± SD (n = 3). *p<0.05 vs. MI; **p<0.05 vs. hMSCs.

Cardiac fibrosis (%) was assessed at 4 weeks following MI (**Fig. 5A, 5B**). Masson-trichrome staining showed a significant decrease in cardiac fibrosis in the hiPSC-CMs (12.9±1.1) transplanted group when compared to the MI (31.1±2.04) or hMSCs (22.7±1.6) groups (**Fig. 5B**). We did not observe any fibrosis in no-MI group. These results suggest that transplantation of hiPSC-CMs attenuates adverse cardiac remodeling.

### Expression of cardiac markers by engrafted hiPSC-CMs in the infarct heart

**Figure 6:**
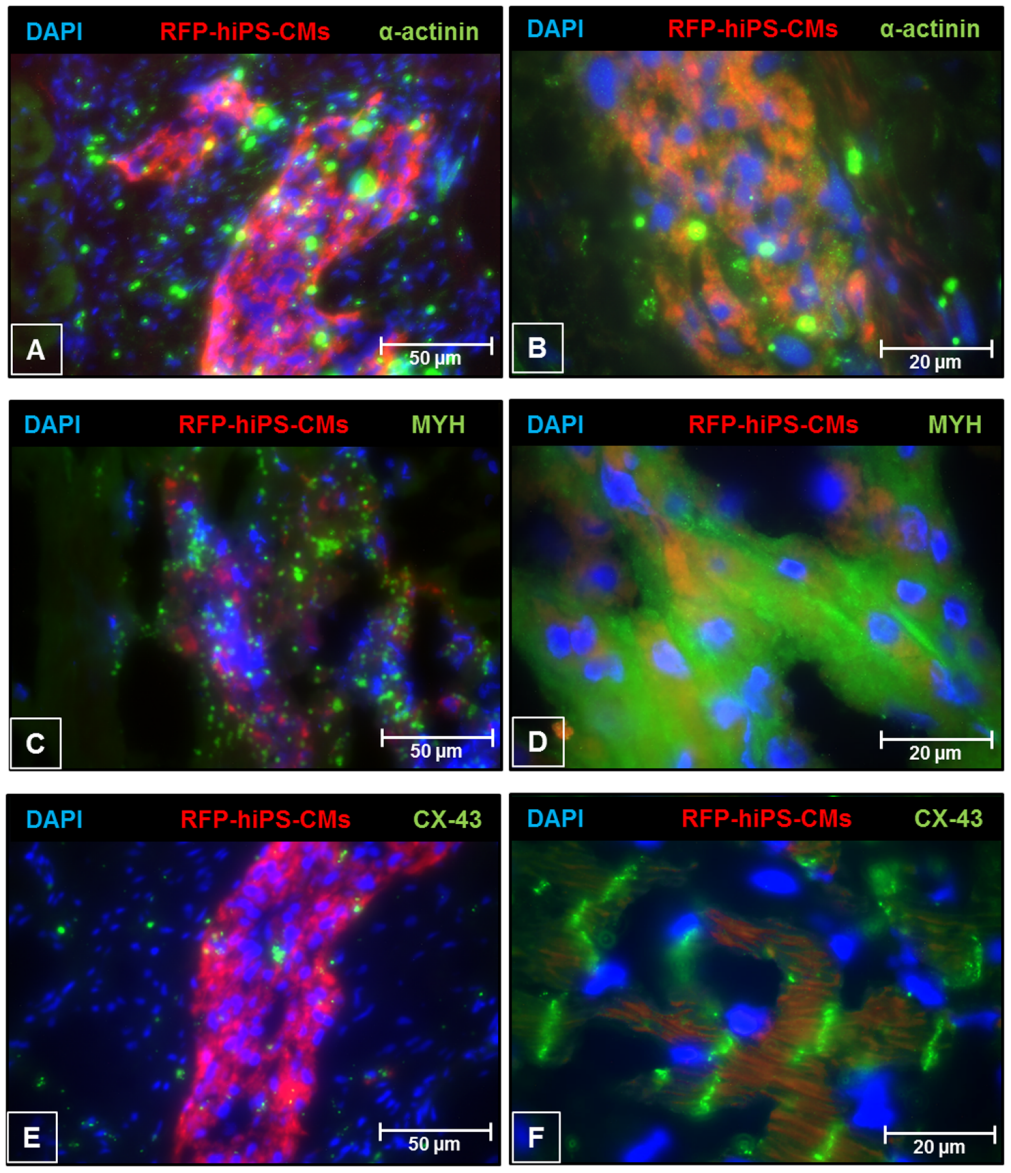
Immunofluorescence imaging of cardiac markers in engrafted hiPSC-CMs at 4 weeks following MI. (**A, B**) α-actinin (**C, D**) Myosin heavy chain (MYH) and (**E, F**) Connexin-43 (Cx-43) in two regions of LV myocardium at different magnifications.

Four weeks following stem cell transplantation, engrafted hiPSC-CMs were visualized by RFP fluorescence. Immunofluorescence staining revealed that the engrafted cardiomyocytes expressed α-actinin, myosin heavy chain and connexin-43 (**Fig. 6A–F**). This data demonstrates that the engrafted cells were able to express cardiomyocyte markers including gap junctional marker connexin-43 in the infarcted heart tissue.

## Discussion

The findings of this study demonstrated that both hiPSC-CMs and hMSCs therapy significantly enhances the recovery of cardiac function at four weeks following myocardial infarction, when compared to MI group. The current study further demonstrated the engraftment of hiPSC-CMs in the infarct heart leading to decreased cardiac fibrosis and superior improvement in cardiac function when compared to hMSCs group. The engrafted cells, previously labeled with SPIO particles were tracked and visualized by cardiac MRI and immunohistology respectively. Our study showed that hiPSC-derived cardiomyocytes were able to express key cardiac markers, such as α-actinin and the gap junction protein connexin-43, both *in vitro* and *in vivo*, thus demonstrating their progression towards a functional cardiomyocyte phenotype.

Bone marrow derived mesenchymal stromal cells (MSCs) have shown improvement in LV function following acute MI [24], [25]. Rose et al demonstrated that murine MSC's display plasticity towards cardiomyocyte lineage while retaining mesenchymal stem cells [26]. In another study, Yannarelli et al showed that infusion of green fluorescent protein (GFP) MSCs into the murine hearts after acute MI led to variable degrees of cardiomyocyte reprogramming of MSCs and majority of cells co-expressed cardiomyocyte and stromal markers [27]. However, MSCs have generally shown a lower engraftment rate in the ischemic myocardium and their primary beneficial effects appear to be paracrine [28]. Controversy remains as to whether these MSC's differentiate into cardiomyocytes after transplantation in the heart. Therefore, in the present study we used human inducible pluripotent stem cells derived cardiomyocytes (hiPSC-CMs) to compare its efficacy with hMSCs in improving the cardiac function and attenuating cardiac remodeling in an acute MI model.

To date, several groups have investigated the potential effectiveness of using numerous iPSC derived therapies to attenuate the negative outcomes associated with post-MI cardiac remodeling. Mauritz et al used iPSC derived cardiovascular progenitor cells to significantly decrease the end-diastolic and end-systolic volumes (EDV, ESV) following acute MI [29]. Further, treated animals showed a significant increase in wall thickness and the percentage of viable myocardium, as well as a significant decrease in infarct size two weeks following MI [29]. Another study showed a significant decrease in LV systolic wall thickness at four weeks following transplantation of iPSC-derived bioengineered myocardium (BM) in a rat model of myocardial infarction [21]. Implantation of iPSC-derived BM also attenuated the progression of fibrosis, when compared to untreated (No-MI) animals. In a similar study Carpenter et al showed efficient cardiac differentiation of human iPS cells to cardiomyocytes and reduced remodeling of the heart after ischemic damage [30]. Most recently, it has been shown that transplantation of human induced pluripotent stem cell-derived cardiomyocytes (hiPSC-CMs) cell sheets improved cardiac function and decreased LV remodeling [31]. Similarly, our present study demonstrated significant improvement in cardiac function and decreased cardiac fibrosis in hiPSC-CMs transplanted group, when compared to MI group.

MRI is one of the non-invasive tools to accurately visualize SPIO-induced hypointense regions within the LV myocardium, thus allowing tracking of hMSCs and hiPSC-CMs engraftment *in vivo*. MR imaging showed engraftment of hiPSC-CMs in the infarct myocardium. This data was further supported by Immunohistological staining of cardiac tissues for RFP and cardiac markers. The hiPSC-CMs were able to engraft and align with native cardiomyocytes in the infarct heart. Moreover, the engrafted hiPSC-CMs stained positive for mature cardiomyocyte markers sarcomeric α-actinin, myosin heavy chain and connexin-43 in the infarct heart. The results from our present study strongly suggest that hiPSC-CMs transplantation leads to regeneration of infarct myocardium. The transplanted hiPSC-CMs were positive for connexin-43, indicating electrical coupling and integration with the native heart. However, further studies are needed to establish electro-mechanical coupling *in vivo* by optical mapping studies which is beyond the scope of the present study. We did not observe any teratoma formation after hiPSC-CMs transplantation.

On the other hand, adverse cardiac remodeling causes fibroblast proliferation that replaces dead cardiomyocytes with scar or fibrotic tissue, which results in heart failure. Our results demonstrated attenuation of cardiac fibrosis in both hiPSC-CMs and hMSCs groups compared to MI group. However, the hiPSC-CMs group showed a significant reduction in cardiac fibrosis when compared to hMSC group. Therefore, we postulate that the attenuation of cardiac fibrosis by hiPSC-CMs could be attributed to autocrine and paracrine factors released by the transplanted cells. Previous studies have attributed the attenuation of cardiac fibrosis due to paracrine factors released by MSCs [32], [33]. However, the factors released by hiPSC-CMs compared to hMSCs need further investigation.

## Conclusions

In summary, this is the first study to compare hiPSC-CMs with hMSCs therapy for treating acute MI. Our findings strongly suggest that hiPSC-CMs are equally potent when compared to hMSCs in improving cardiac function and superior in attenuating fibrosis. However, the exact mechanism by which hiPSC-CMs attenuated cardiac fibrosis needs further investigation. Transplanted hiPSC-CMs integrated with the host myocardium and expressed mature cardiomyocyte markers indicating electromechanical coupling of engrafted cardiomyocytes. Our results suggest that hiPSC-CMs may possess cogent autocrine and paracrine mechanism for treating MI and may have a potential clinical impact in future for treating patients with acute myocardial infarction.

## Supporting Information

S1 Video Human inducible pluripotent stem cells derived cardiomyocytes were cultured on gelatin coated plates. Human iPSC-CMs demonstrated spontaneous beating at one week of culture in regular cell culture plate maintained at 37°C and 5% CO_2_.
(MOV)
